# The Complete Genome Sequence of *Trueperella pyogenes* UFV1 Reveals a Processing System Involved in the Quorum-Sensing Signal Response

**DOI:** 10.1128/genomeA.00639-17

**Published:** 2017-07-20

**Authors:** Vinícius da Silva Duarte, Laura Treu, Stefano Campanaro, Roberto Sousa Dias, Cynthia Canedo da Silva, Alessio Giacomini, Viviana Corich, Sérgio Oliveira de Paula

**Affiliations:** a Department of Microbiology, Universidade Federal de Viçosa, Viçosa, Minas Gerais, Brazil; b Department of Agronomy Food Natural Resources Animals and Environment, University of Padova, Legnaro, Italy; c Department of Biology, University of Padova, Padua, Italy; d Department of General Biology, Universidade Federal de Viçosa, Viçosa, Minas Gerais, Brazil; e Department of Environmental Engineering, Technical University of Denmark, Lyngby, Denmark

## Abstract

We present here the complete genome sequence of *Trueperella pyogenes* UFV1. The 2.3-Mbp genome contains an extremely interesting AI-2 transporter and processing system related to the quorum-sensing signal response. This specific feature is described in this species for the first time and might be responsible for a new pathogenic behavior.

## GENOME ANNOUNCEMENT

*Trueperella pyogenes*, formerly *Arcanobacterium pyogenes*, is a worldwide opportunistic pathogen possessing a broad range of virulence factors which have been involved in economic losses due to diseases, like mastitis and metritis, in dairy cows (1, 2). The capability of this species to generate biofilms by growing as a sessile bacterial community is key in determining chronic infections. Biofilm formation, in fact, allows microorganisms to have better resistance in selective environments, which are determined by exposure to antibiotics or the immune defense system (3, 4). In *T. pyogenes*, little is known about the gene expression of the principal virulence factor pyolisin (PLO) *in vivo*; however, its transcription is related to biofilm formation and is positively regulated in the beginning of stationary phase by the response regulator *ploR* (3).

Here, we present the complete genome sequence of *T. pyogenes* UFV1, a strain isolated from a dairy cow affected by metritis that was raised in a dairy farm (Viçosa, Minas Gerais, Brazil). This strain was used in these studies of biofilm control and as a phage host.

DNA extraction was performed using the protocol described by Pospiech and Neumann (5) and sent to MR DNA. Sequencing was performed with the Illumina MiSeq platform using paired-end (PE) reads (2 × 300 bp) and Nextera library preparation. The sequences were assembled *de novo* using the CLC Genomics Workbench software (version 9.5), and scaffolds were ordered and oriented using strain *T. pyogenes* TP6375 as the reference with CONTIguator (6). The RAST server (7) and the NCBI Prokaryotic Genome Annotation Pipeline (PGAP) were used for gene finding and annotation.

After quality filtering and merging of the overlapping PE reads, a total of 2,626,752 sequences, with an average length of 314 bp, were obtained, providing nearly 360-fold genome coverage. The assembly resulted in 23 scaffolds, with a total length of 2,333,212 bp and average G+C content of 59.5%. Alignment of the scaffolds with the reference strain suggests they are arranged in a single circular chromosome. PGAP predicted 2,199 genes classified into 2,142 coding sequences (CDSs), 2 clustered regularly interspaced short palindromic repeat (CRISPR) arrays, 2 rRNAs, 51 tRNAs, 4 noncoding RNAs (ncRNAs), and 40 pseudogenes. Additionally, 47% of the CDSs were assigned to 308 subsystems using the RAST server.

The genome analysis revealed a new feature of *T. pyogenes*, the *lsrACDBFGE* operon, which is related to the quorum-sensing signal response AI-2, described here for the first time for this species. This system is known to mediate interspecies communication (8), and we hypothesize it is involved in the newly described pathogenic ability of *T. pyogenes*, since biofilm formation and virulence factors are regulated by this mechanism. Additionally, a broad range of virulence factors were found specifically related to genes that confer resistance to antibiotics (vancomycin, streptothricin, fluoroquinolones, beta-lactams, and aminoglycosides) and cell wall attachment (*nanH*, *nanP*, *cbpA*, *fim*, and *pl*o).

In this work, we present the complete genome sequence of *T. pyogenes* UFV1, which will serve as a phage host and a new model in order to understand the role of quorum sensing in this species.

### Accession number(s).

This whole-genome shotgun project has been deposited at DDBJ/ENA/GenBank under the accession number MVGS00000000. The version described in this paper is version MVGS01000000.
